# The occurrence of individual slow waves in sleep is predicted by heart rate

**DOI:** 10.1038/srep29671

**Published:** 2016-07-22

**Authors:** Armand Mensen, Zhongxing Zhang, Ming Qi, Ramin Khatami

**Affiliations:** 1 Clinic Barmelweid, Aargau, Switzerland

## Abstract

The integration of near-infrared spectroscopy and electroencephalography measures presents an ideal method to study the haemodynamics of sleep. While the cortical dynamics and neuro-modulating influences affecting the transition from wakefulness to sleep is well researched, the assumption has been that individual slow waves, the hallmark of deep sleep, are spontaneously occurring cortical events. By creating event-related potentials from the NIRS recording, time-locked to the onset of thousands of individual slow waves, we show the onset of slow waves is phase-locked to an ongoing oscillation in the NIRS recording. This oscillation stems from the moment to moment fluctuations of light absorption caused by arterial pulsations driven by the heart beat. The same oscillating signal can be detected if the electrocardiogram is time-locked to the onset of the slow wave. The ongoing NIRS oscillation suggests that individual slow wave initiation is dependent on that signal, and not the other way round. However, the precise causal links remain speculative. We propose several potential mechanisms: that the heart-beat or arterial pulsation acts as a stimulus which evokes a down-state; local fluctuations in energy supply may lead to a network effect of hyperpolarization; that the arterial pulsations lead to corresponding changes in the cerebral-spinal-fluid which evokes the slow wave; or that a third neural generator, regulating heart rate and slow waves may be involved.

While the precise function of sleep remains one of the greatest mysteries to neuroscience, the past several decades has provided a large body of data and subsequent theories about the underlying processes and mechanisms involved. One of the most fruitful aspects of this research have been discoveries about the structure of slow-wave or non-REM (rapid eye movement) stage 3 sleep; primarily characterized by the appearance of slow oscillations in the electroencephalographic (EEG) recordings in the frequency range 0.5 – 4 Hz with large, negative-going amplitudes generally exceeding 80 uV. At a cellular level slow wave oscillations are associated with down-states in which neurons are hyper-polarized and virtually silent that alternate with up-states of sustained depolarization. Most studies have been concerned with the large-scale changes in the brain that underly the non-rapid-eye-movement (NREM) state and have regarded the individual slow waves as spontaneous events, with no immediately-apparent or consistent cause^1^,^2^,^3^. Indeed, models of the thalamo-cortical system show that, given certain plausible assumptions of the model, changing parameter constants will inevitably lead to synchronized down and up-states which would correspond to slow waves in the local field potential, and hence the EEG^4^. The most influential, and necessary parameter change is the increase of the potassium leak conductance of the individual neurons, a direct effect of the reduction to the neuromodulators acetylcholine and norepinephrine, which leads to a drop in the resting membrane potential during NREM^5^. Several studies have demonstrated that certain factors can predict aspects of individual slow-waves; most notably, use-dependency during wakefulness^6^,^7^; but also preceding beta-range activity^8^. However, there has been little research examining factors which share a close temporal relationship to individual slow waves^9^. While the necessary background conditions take the probability of a slow wave up from essentially zero, here we report that the chance of an individual slow wave occurring, at precisely the time that it does, is related to individual heart beats.

The electrical activity of slow waves invoke distinct metabolic requirements and energy demands. While the sustained neuronal firing during up-states leads to increased metabolic rates, the neuronal inactivity of off-periods are associated with low energy expenditures. Due to neuro-vascular coupling, increased neuron activity will induce a haemodynamic response to compensate with the higher energy demand. Sleep-state related shifts of oxygenation, blood volume, blood flow and blood volume has been shown at a system level by using various imaging techniques (such as PET, SPECT and fMRI) and at the level of neuronal columns in the cortex^10^,^11^,^12^. Thus, considering two markers of slow wave sleep, an electrophysiological and a metabolic/hemodynamic marker, may provide new insights into the changes of background conditions linked to single slow waves. Near-infrared spectroscopy (NIRS) measures are primarily sensitive to haemodynamic changes and do not suffer from the same restrictions as fMRI or PET environments impose. NIRS can be recorded using relatively small, mobile devices which can be used at the bed side, and only require a small patch to be placed on the skin containing a light source and at least one light sensor. Moreover, NIRS is sensitive to changes in both oxy- and deoxyhaemoglobin, and as such, blood volume measures can also be obtained in parallel. This makes the combination of NIRS and EEG measurements an ideal tool for examining the haemodynamic response in relation to events during sleep^13^,^14^.

## Results

### NIRS

**Figure 1:**
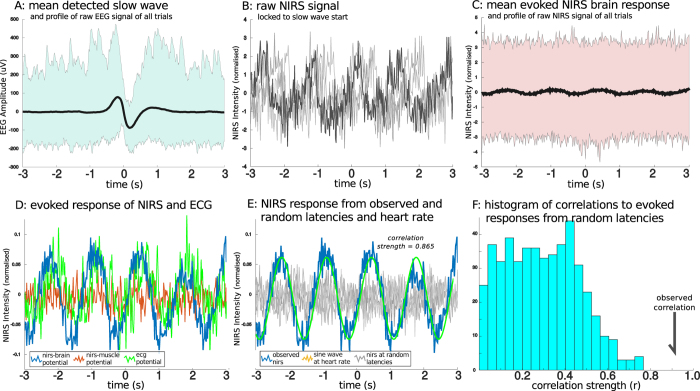
Single Night Processing and Statistics. The basic stages of data processing from raw signal to statistics evaluation of a single subject are outlined. (**A**) the mean of all slow waves, locked to the downward zero crossing is shown in black with the outline of the raw signals shown in the background. (**B**) the first four trials of raw near-infrared spectroscopy (NIRS) signal (alternative-current) from the sensor over the prefrontal cortex, locked to the slow wave onset in various shades of grey. Notice that the oscillating pattern related to the heart-rate is already apparent in the raw signal. These values undergo a linear detrending, but no further signal processing steps are applied to the data. (**C**) the NIRS evoked response from all slow waves in relation the outline of the raw signal. (**D**) the final evoked signal, normalized by the mean standard deviation of the raw signal (proportional to the outline of raw NIRS depicted in panel (**C**) from the NIRS sensor of the brain, the NIRS sensor over the bicep muscle, and also the evoked potential of the electrocardiogram measured across the heart. (**E**) the same evoked NIRS response from the brain as in (**C,D**) but with an overlaying generated sine wave with an oscillating frequency corresponding the mean heart-rate from slow wave sleep in orange. Depicted in the gray background is a selection of the evoked responses created using random latencies instead of the slow wave onset. (**F**) a histogram of the pearson correlation coefficients of the evoked NIRS response to the generated sine wave from both 500 randomly selected latencies and the observed correlation of the evoked response when locked to the onset of the slow wave. The individual p-value is the proportion of random correlation coefficients which are greater than the observed correlation.

**Figure 2:**
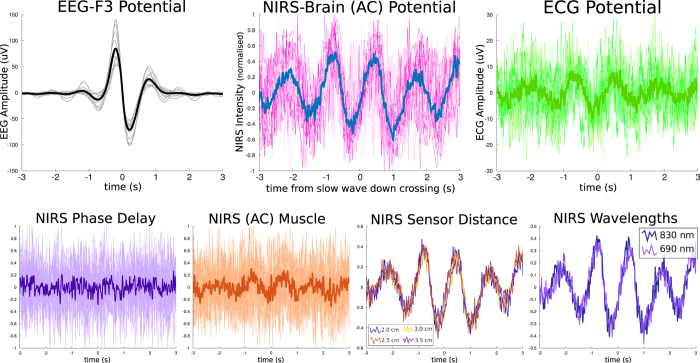
Group Data. Plots of the evoked responses from the different measures captured by the NIRS sensor and also the electrocardiogram measures taken as the mean from all 16 nights of recordings from 8 different participants. (**A**) mean slow wave at the F3 electrode position, the one nearest to the NIRS sensor. A mean of 2288 (se = 103) individual slow waves were detected from each night of sleep recording. (**B**) group mean NIRS potential from the pooled alternating-current (AC) channels of different wavelengths and distances. Twelve of sixteen participants showed a significant individual relationship between the oscillating potential and a sine wave depicting heart rate oscillation. (**C**) mean group evoked potential from the electrocardiogram (ECG) measure. The same oscillating signal is found in 13 of 16 participants but is generally weaker and noisier than the NIRS-AC potential. (**D**) evoked potential of the phase-delay (PD) measure from pooled NIRS channels over the brain sensor showing no such heart-rate dependent oscillations. (**E**) pooled channels for the AC measure of the NIRS signal placed over the bicep muscle, like the ECG potential, still shows the oscillating pattern but much weaker. Here, only 7 of 16 recordings showed a significant oscillation at the individual level. (**F**) the AC-NIRS measure over the brain sensor broken into its contributing channels at different sensor distances corresponding to deeper penetration into the cortex; no differences in sensor distances were found. (**G**) the AC-NIRS measure over the brain sensor broken into its contributing wavelengths. While some participants differ in the strength of the oscillation between wavelengths, there is no one consistent wavelength which performs better over all participants.

Figure 1 shows the processing of a single example individual and their results. Figure 2 shows the group average and individual event-related potentials of the NIRS signal (nERP) when time-locked to the start of the EEG slow wave. As the potential is clearly oscillatory in nature, the single summary statistic calculated was the correlation coefficient to a generated sine-wave; with a frequency corresponding to the individual participant’s heart rate during sleep. Of primary interest was whether the oscillatory nERP was significantly different from that evoked for random latencies which was tested using a permutation approach (see Fig. 1 and methods). The mean correlation coefficient for all participants for the pooled AC signal from the brain sensor was 0.604 (se = 0.066) with 11 of 16 recordings showing a significant individual effect and another participant showing trend levels. Those participants who showed significant individual effects had a mean correlation of 0.746, with a mean p-value of 0.012 and a mean phase delay of 176 ms from the nadir of the sine wave to the start of the slow wave.

When compared with the pooled AC signal for the muscle sensor, only 7 of 16 participants showed significant individual effects with a mean correlation of 0.220 (se = 0.043; mean correlation for significant participants = 0.349; mean p-value 0.016), and a further two showed trend levels of significance. Importantly, no participant showed a significant effect for the muscle sensor that did not also show an effect for the brain sensor. At the group level, the brain sensor oscillation was significantly stronger than the muscle sensor (T_7_ = 4.794, p < 0.001). For the PD measures at the brain sensor, only 2 of 16 participants showed a significant, albeit weak, individual correlation to the sine wave (mean correlation 0.086, se = 0.013). None of the participants showed a significant oscillation for the PD measures from the muscle sensor, nor did the PD measures correlate with AC measures (r = −0.118, p = 0.662).

Given the strong evoked potential for the pooled AC measure for the brain sensor, we investigated whether any particular sensor distance or wavelength contributed to the effect over any other. There were no significant differences at the group level between the two wavelengths, 830 nm and 690 nm (T_7_ = −0.582, p = 0.001), yet neither did the two measures correlate with one another (r = 0.334, p = 0.207). Thus, for some participants one wavelength showed a stronger relationship than the other; but which wavelength specifically, varied between participants. As expected, neither wavelength was significantly different from the pooled AC signal (830 nm: T_7_ = 1.385, p = 0.186; 690 nm: T_7_ = 0.762, p = 0.458). Interestingly, while the 690 nm wavelength showed a higher group mean effect, only 9 of the 16 participants showed a significant individual effect (a further 3 showed trend levels), while 12 of the 16 participants showed individual significance for the 830 nm signal. It should also be noted that the pooled signal showed a stronger effect than either wavelength separately (830 nm correlation = 0.523, se = 0.076; 690 nm correlation = 0.571, se = 0.063), likely owed to the reduction of noise through pooling while capturing essentially the same signal.

Each channel, recorded from different distances to the light source, also showed highly consistent results. For each distance, the same 12 of 16 participants showed significant individual results, albeit having slightly different correlation and p-values per channel. A one-way repeated measures analysis of variance showed no significant differences between any of the distances (F_1.9, 28.68_ = 1.066, p = 0.348). However, the nearest channel, and thus the one with the least light penetrating the cortex, showed the weakest mean correlation values (2 cm mean = 0.523, se = 0.076; 2.5–3.5 cm mean = 0.606, se = 0.057).

Given that the timing of different phases of the slow wave, the start, peak negativity and subsequent up peak are related to one another, it is possible that the oscillation is not necessarily synchronized to the start of the slow wave, but rather to its peak or upward point. We re-ran the analyses with the pooled AC NIRS signal linked to the timing of the local minima of each slow wave (the timing of the highest neural synchronization), and also the upward zero crossing (the timing of re-activation of the cortex). The mean correlation value locked to the peak of the slow wave was lower than that for the start of the slow wave (mean correlation to the peak= 0.554, se = 0.077; mean correlation the slow wave onset = 0.604, se = 0.066); however this difference was not found to be significant (T_7_ = 0.924, p = 0.370). The NIRS oscillation locked to the negative peak correlated significantly better in the brain than in the muscle (T_7_ = 4.633, p < 0.001). The same trend was found for NIRS locked to the upward zero crossing which again showed a lower effect (mean correlation = 0.530, se = 0.61) compared to both the start of the wave and the peak synchronization but which also was not significantly different from the oscillation strength to the start of the slow waves (T_7_ = 1.574, p = 0.136).

### ECG

Given the finding from the NIRS data that clearly related the nERP oscillation to the individual participant’s heart rate, we were interested to see whether the evoked response from the ECG (eERP) would show the same oscillation. The eERP also showed a clear oscillation (see Fig. 2; top right) and had mean correlation of 0.508 (se = 0.060) to the generated sine-wave. Although the strength of oscillation was lower than the mean for the AC NIRS on the brain (0.604), 13 of the 16 participants showed a significant individual effect with a mean phase delay of 66 ms (se = 35 ms) from the nadir of the evoked response to the start of a wave. Of note is that the one participant that displayed an opposing phase oscillation of the pooled NIRS signal, did show a significant ECG evoked potential. At the group level, the ECG response was not found to be significantly lower than the AC response at the brain sensor (T_7_ = 1.789, p = 0.094), and was found to significantly correlate (r = 0.641, p = 0.007), such that the strength of the participants’ NIRS responses could already be predicted using the evoked ECG response. Surprisingly, the same relationship was not true between the ECG potential and the AC measure from the muscle electrode where the relationship was significantly better for the ECG measure (T_7_ = −4.919, p < 0.001), and were not predictive of one another (r = 0.389, p = 0.137). While there is only one oscillatory component in the NIRS data, the ECG signal consists of several closely tied components: the fast components of the QRS complex, and the slower P-wave just after the QRS complex and the smaller T-wave just prior to the subsequent QRS complex. Despite the finding that the ECG evoked potential is a slow oscillation, this may still be more related to the fast components that are not perfectly synchronized and as such the oscillation represents more of probability curve, or the evoked potential could represent a tighter relationship to the slow components directly. In order to examine which of these components is the more likely contributor to the evoked potential, we re-ran the same analysis using the generated sine wave for ECG data under two different filters in attempt to isolate the primary contributor: firstly with a bandpass filter between 0.3 and 10 Hz; then again using a bandpass filter between 10 and 40 Hz. The results here were clear, if we isolate only the QRS complex using a high bandpass filter, only a single participant was found to have a significant oscillation pattern related to the heart rate. On the other hand, even if we attenuated the effect of the QRS complex, and thus enhanced the contribution of the slower components, the P and T-waves, using the lower bandpass filter the same 13 of 16 participants showed a significant individual oscillation with an even higher mean correlation value (0.606, se = 0.064 versus 0.508, se = 0.060).

### Sleep and slow wave parameters

Each participant had a relatively normal sleep pattern with a participant mean of 6.6 hours (se = 13 minutes) spent asleep each night. Of the time asleep, the mean percentage of time spent in N1 was 8.2% (se = 1.6%), N2 was 44.7% (se = 2.0%), N3 was 30.4% (se = 2.1%) and finally REM was 16.3% (se = 1.4%). It was not feasible to measure the latency to N1 since there was often no clear point where the participant would have initiated their attempt to sleep as the lights may have been turned off while the NIRS device was still calibrating. Thus, sleep latencies were considered from the first epoch scored as N1. Latency to the first N2 stage were fairly short with a mean of 3.6 minutes (se = 1.0), and subsequent N3 latency for the group was 12.2 minutes (se = 2.5), with REM latency understandably more varied with a mean of 106.2 minutes (se = 13.6). Surprisingly, almost none of these measures showed significant differences between the baseline and recovery night after chronic sleep deprivation except for time spent awake after initial N1 (mean difference 1.6% less for recovery night; T_7_ = 2.556, p = 0.038). Yet all measures had mean differences in the expected direction with decreased time spent in N1, increased time in REM sleep and reduced latencies to all stages. The SWA toolbox^6^, detected a mean of 2288 waves (se = 103) per night, which corresponded to a mean of 7.8 waves per minute (se = 0.7) for stages N2 and N3. Detected waves had a median amplitude of −104.6 uV (se = 6.1), and deviation in amplitude of 28.9 uV for each participant (se = 1.4).

## Discussion

Here we found, for the first time, an ongoing oscillation in the NIRS AC signal that was time-locked to individual EEG slow waves. The origins of this oscillation at the single trial level suggest it is the reflection of the well-known heart-rate ‘artifact’ present in most NIRS recordings; often removed during the preprocessing stages of NIRS experiments. This assumption is supported by the additional finding of the oscillation in the NIRS signal from the sensor over the bicep muscle; albeit with a weaker correspondence. Moreover, this oscillating signal can also be detected by time-locking the raw ECG signal to the start of the slow wave. The nature of this heart-rate oscillation in NIRS has been related to light absorption changes of the moment to moment fluctuations in blood flow/volume and pressure in arterial vessels induced the pressure wave generated by the heart beat^15^,^16^,^17^,^18^. The finding that all channels at various distances find essentially the same signal at the same strength (relative to the noise of the individual channel), also supports that this signal comes from the superficial haemodynamics and not the cortical layers. While the oscillation is also statistically related to the peak and end of the slow waves, the link to the onset of the down-state is the most consistent both in terms of the strength of the correlation and across participants. This finding is somewhat surprising since the precise timing of the onset of the slow wave is relatively arbitrarily set to the downward zero-crossing, and as such this timing value should be more prone to noise compared to the more precise timing of peak neural synchronization given by the negative peak of the slow wave.

The current experiment finds only the correlation between the NIRS signal and generation of slow waves. The ongoing nature of the oscillation, and the relative rarity of the slow wave suggest that it is the slow wave that is dependent on the oscillation. However, whether it is some direct property of the oscillation and its consequences that leads to the initiation of the cortical down-state, or whether there is a third, yet unknown, mechanism leading to both remains speculation at this stage. One possible explanation is that once the background conditions for slow wave sleep are met (the right ‘cocktail’ of neurotransmitters and neural activity, adequate energy supply etc^4^,^19^,^20^,^21^), cortical neurons would eventually go into down-states in any case and the fluctuation in blood flow provides the small ‘push’ they need to do so at their actual timing given this bi-stable state^22^. This may happen in one of two ways: the heart-beat can act as a neural stimulus which evokes a down-state in the same way as has been shown for an auditory, sensory^23^, or even magnetic stimulation^24^. Such an account is supported by a recent study examining how the heart-rate is linked to various ongoing oscillations found in the EEG, and how these are altered from wake to deep sleep^9^. Similar to our approach, that study event-locked the EEG to ECG in periods of slow-wave sleep. Yet, they examined the timing of the R-wave in relation to the positive-going zero-crossing of individual slow waves and found an increase in R-wave occurrence during this time.

The second alternative stems from the finding that neurons in a cortical column may enter a hyper-polarized, sleep-like state when their resources are depleted; possibly as a protective mechanism^25^. The relatively small effect of low regional blood flow between heart beats on a few critical neurons could create a network effect of reduced but synchronized firing that spreads as a regional (and then possibly globally), traveling slow wave over the cortex^1^. However, we find that the lowest light intensity, indicative of the highest absorption and blood flow occurs just prior to slow wave initiation. Thus, for this hypothesis to be plausible there would have to be a substantial delay (>500 ms) between the start of the local down-state and its visibility on the EEG. While such a delay weakens the hypothesis, it is not without some support as external stimuli such as sounds or touch also tend to evoke a slow wave only after 500 ms^23^.

While neuro-vascular coupling sufficiently explains changes in blood flow and oxygenation as a consequence of neuronal activity, a potential function of arterial pulsations related to slow waves have not yet been considered. It has been shown in animals and humans that arterial pulse waves induced by each cardiac cycle travel along penetrating intracortical arteries to drive the para-vascular CSF pulsations and thus promote CSF-brain interstitial fluid (ISF) exchange^26^,^27^. This CSF-ISF exchange allows clearance of accumulated metabolites from the brain. Given our current finding that vascular pulsations facilitate the occurrence of slow waves and that slow wave sleep increases the para-vascular space by 60%, metabolic clearance of the brain by arterial pulsations is much more efficient (due to favorable convective conditions) during slow-wave-sleep^28^. This idea is compatible with the restorative function of sleep with slow-wave-sleep as the substrate of homeostatic drive and sleep need.

Additional findings for the functional link between slow wave initialization and the heart come from the finding that the theta-rhythm of neural activity in the hippocampus is also correlated with heart-rate variability^29^,^30^,^31^. While during wakefulness there is a clear neural pacemaker for the heart in the brain stem^32^, these studies find that, during REM sleep, there is significant phase-locking between the hippocampal theta-rhythm and the ECG. These authors offered two alternative hypotheses. The first was that the spontaneous theta-rhythm directly contributed to the heart rhythm during REM sleep, although with an unclear mechanism, or that a third neural source, with outputs to both the hippocampus and sympathetic nervous system simultaneous controlled both. Since our data are also only suggestive of a correlation between slow waves and the heart-rate, both of these hypotheses are also possible. Given the consistency of the theta-rhythm, and fact that it is faster than the heart-rate, it seems more natural to insist on the causal direction from brain to the heart. However, slow waves generally occur much more sporadically and thus such a tight neural mechanism seems less likely. Given that our initial posited mechanism of spontaneous energy constraints from blood flow requires no third causal entity, a mechanism whereby slow waves may affect the heart rhythm or that there exists a neural generator that generates slow waves and controls heart rhythm seems superfluous. Moreover, the previously found relationship between theta-rhythm and heart-rate could just as well be reinterpreted under the same energy constraints as we suggested earlier in that the theta-rhythm could also be driven by the local fluctuations in heart-rate.

The unexpected nature of this finding raises the question of why this relatively straightforward relationship had not been previously uncovered. While NIRS is a fairly novel technique, we also show that the relationship can be discovered using ECG and EEG measurements alone; both having been used for over a century. It also certainly not the case that some relationship between the two measures during sleep had not previously been hypothesized since changes in overall heart rate and its variability are highly predictive of sleep stage^33^,^34^,^35^. The initial possibility is that others have indeed explored the relationship, could not find one, and as such we have falsely rejected this null hypothesis. This seems unlikely to be the case the relationship is confirmed at the individual and group levels of analysis using independent measuring techniques of the voltage-based ECG signal and the light absorption technique of NIRS. Moreover, no filtering or significant signal processing techniques were applied to the raw data from the original recordings and obtaining the nERP consists of merely taking the mean activity from the raw data of time-locked trials. One major reason for the lack of earlier discovery is possibly that the detection and analysis of individual slow waves is a relatively recent trend, and that openly available tools were lacking. Only within the last decade or so have we found that studying the parameters of individual waves could be a much more accurate and explanatory method than classical methods such as mean power in the low-frequency spectra^23^. Another contributing factor is that the oscillation found for the evoked potential in the ECG is relatively weak and noisy and certainly not immediately obvious from an examination of the raw data. As such it also requires increased accuracy in the timing of the slow wave properties, and also many exemplars in order to improve the signal-to-noise ratio.

Although our findings have novel implications in understanding the mechanism of individual slow wave generation, future work should reproduce this result. Ideally, this finding could be extended by using high-density recordings of both the EEG and NIRS measurements. In this way, the small fluctuations in blood flow over the cortex could be tracked along with the traveling nature of the slow wave^1^, where the best correspondence between the two would provide evidence towards the causal nature of the effect. For example, we would expect that the strongest link between the NIRS oscillation and slow wave would be found at the channels where the slow wave originates, and not where the slow wave is already large enough to be detected globally. While we found that sensor distance was irrelevant to the oscillation, which of the two wavelengths measured was more sensitive to the oscillation depended on the participant. Since this possibly reflects the variation in sensor positioning relative to individual anatomy, it would useful to examine different sensor positions relative to the individual MRI; looking closely at both cortical folds and location and direction of large arterial vessels. Future work should also attempt to distinguish the two plausible explanations put forth, the spontaneous energy constraint, or the common neural generator models. One way to do this would be to examine the relationship between NIRS and slow waves in heart transplant patients whose heart rhythm is controlled via a pace-maker and not a neural source; if these patients still show an oscillating nERP it would rule out the common neural generator hypothesis. Moreover, patients with cardiovascular disease are likely to show a weaker variability in blood flow, and if the energy constraint model is correct we would expect the level of fluctuation to be proportional to the strength of the nERP oscillation.

## Methods

### Participants

8 health high school students (4 female, ages 18–19 years old) participated in this study. None of the participants had any sleep disorders, cardio-vascular disorders, diabetes, obesity, or any psychiatric issues. The study was approved by the local ethical committee of the Canton Aargau, Switzerland and experiments were performed in accordance with its guidelines and regulations. All participants were given written and verbal instructions as to the nature of the recordings and were required to sign a consent form.

### Sleep Recording and Processing

Each participant spent two nights at the sleep center; a baseline night and a recovery night following 5 nights of partial sleep restriction of no more than 5 hours of sleep. Actimetry was used to monitor their sleep-wake rhythm prior to coming to the clinic, as well as during the time of their sleep restriction. During the night at the clinic, sleep was monitored using a full polysomnography (PSG) which included 6 EEG electrodes on the scalp as well as two reference EEG electrodes on both the mastoids and a grounding electrode. Scalp electrodes were placed using the 10–20 system at F3, F4, C3, C4, O1, and O2. Moreover, two electrodes on the chin measured the participants’ electromyography and two chest electrodes measured their electrocardiogram (ECG). Signals were amplified and recorded using the XLTEK (Natus Neurology, Excel-Tech Ltd., WI, USA) at a sampling rate of 200 Hz and exported into Matlab (2015a, The MathWorks, Inc., Natick, Massachusetts, United States.) for further analysis using the sleep wave analysis toolbox^36^. Sleep stages were scored in 30 second epochs using the AASM scoring guidelines and marked for arousals and EEG artifacts^37^.

The detection and analysis of individual slow waves was performed using the semi-automatic algorithms openly available in the sleep-wave-analysis toolbox for Matlab. Only parts of the EEG time series scored as N2 or N3 were input into the toolbox for slow wave detection, not including arousal or artifact sections. The algorithm used the negative envelope of all 6 channels, bandpass filtered between 1 and 4 Hz, and treated this as a canonical wave onto which to apply slow wave criteria examining the downward and subsequent upward crosses of the signal. A portion of the signal was marked as low wave which a negative amplitude more than 5 standard deviations from the median negative activity; a negative wavelength between 250–1250 ms; and a positive slope in the 90^th^ percentile. Whether an individual channel was counted as containing a slow wave was determined by cross-correlating the negative envelope at the point where the preliminary criteria were met with each individual channel with a maximum phase delay of 100 ms. A channel had to correlate, at some given phase, to at least an r-value of 0.9 to be included. Additionally, the minimum peak of the individual channel had to be in the negative range with an amplitude of at least 10% of the amplitude criteria. If no individual channel met the criteria, the entire slow wave was considered a false positive and removed. Once the waves were automatically detected, we used the toolbox to manually check through all the waves and delete waves which were clearly false positive as well as manually add waves that the algorithm had missed. In this way, we ensured the maximum number of true slow waves for each participant.

### NIRS Recording and Processing

NIRS was recorded using a frequency domain NIRS system (Imagent, ISS, Champaign IL, USA), using two probes. Each probe contains one light detector and four sources, with various source-detector distances of 2.0, 2.5, 3.0 and 3.5 cm^16^. One sensor was placed on the left of the forehead, just under the participants’ hairlines, thus covering a large portion of the left dorsolateral prefrontal cortex, while the other was placed over the left bicep muscle of the arm as a control measure. The sampling rate of the NIRS recording could be altered according to the desired single-to-noise trade-off and varied between participants of either 36, 42 or 52 Hz with the majority of participants recorded at 52 Hz. The NIRS machine and PSG recordings were synchronized using a single coaxial trigger cable which sent a signal whenever the NIRS machine was recording data. Both the start and end triggers from the NIRS device were used to accurately translate the event triggers for slow wave starts such that any small deviations of the reported sampling rates from either the NIRS or EEG recording devices was also taken into account and corrected for.

The data from the NIRS device, imported as a text file into Matlab, consisted of raw data from each sample in rows from each of the 24 individual measures from each of the two sensors in columns. From each sensor, the direct current (DC), alternating current (DC), both light intensity measures, as well as the phase delay (PD) for each of the 4 sensor distances were reported for both light emitted at 690 nm and 830 nm wavelengths. Initially, all the data from AC measures were pooled from the brain and muscle sensors separately. The same was done for the PD measures. After initial inspection of the results, the AC signal was broken down into its corresponding sensor distances and wavelengths in order to examine whether any particular part of the pooled AC signal was more informative over another. Event-related potentials were taken for the NIRS data time-locked to the start of a slow wave defined by the time of the downward zero-crossing of the wave in the filtered EEG signal. No filters were used on the NIRS data to avoid unforeseen effects of preprocessing steps and specific manipulation of the NIRS signal. Three seconds prior and post to the start of the slow wave were taken from the NIRS signal, then each trial was then independently detrended using a simple linear filter (this step does not alter the data in any considerable way except for removing the inherent slow drift portion of the NIRS signal). The mean of all trials was then calculated to give the event-related potential of the NIRS signal (nERP). In order to compare the signals from different wavelengths of sensor distances directly with each other, we normalized the resulting nERP by dividing the signal by an estimate of the amount of noise (the mean standard deviation of all the raw trials). This normalization step was only done for visualization purposes, as shown in the figures, but the non-normalized signal was used for all statistical analyses. The typical step of removing the heart-rate artifact, using a least-squares regression algorithm, was explicitly not done since we initially suspected that any effect would mean to zero given that we had several thousand trials per participant, and also, once the results were examined it was suspected that the same pulses stemming from the heart rate may be responsible for the nERP uncovered^15^.

Since the heart artifact is clearly visible in the raw NIRS signal, and that the nERP is simply created by an average of the signal, it is theoretically plausible that the oscillatory nature of the nERP is the reflection of any small but essentially random bias in the distribution of events in relation to the phase. Although made increasingly less likely to show up given the vast number of trials in making the ERP, one can imagine the scenario that even with 2000 trials, the mean of 1999 is perfectly reduced to zero activity, meaning the oscillatory nature of the next trial would determine the shape of the entire nERP. Thus, a single statistical measure is required which captures the oscillatory nature of the nERP while taking into account that random latencies may also generate an oscillatory response. The simplest single measure to test the oscillation phase and strength is the correlation between the signal and a generated sine wave. Furthermore, the frequency of the sine wave was adjusted to correspond to the mean heart rate during N3, and the starting phase of the wave was such that the local minima corresponded to the start of the slow wave (time point zero). In order to allow for some delay between the two signals, we cross-correlated the two with a maximal phase delay of half the wavelength of the slow wave.

This process resulted in two measures of interest; the Pearson correlation coefficient and the phase delay corresponding the peak of the correlation of the signals. To test for individual significance, a permutation approach was used with the null hypothesis that the nERP for random latencies would show similar properties to the nERP corresponding to the start of the slow wave. Thus, the same analysis (cross-correlation to the generated sine-wave), was repeated for 5000 newly created datasets corresponding to the nERP of random time points in the EEG independent of the actual slow wave start times. The p-value for individuals corresponded to the position of the actually observed correlation in the empirical distribution of correlations from random time points (see Fig. 1). Importantly, this statistical method is actually likely to be conservative and underestimate the effect. Firstly, it does not take the amplitude strength of the nERP into account, which is generally substantially higher than the nERP of randomly selected times. Secondly, while the phase delay of the observed signal is fairly consistent the correlation coefficient of the randomly timed signals is the maximum across several phase delays to the sine wave; thus the consistency of the phase delay to the sine wave across participants is also not taken into account for the individual significance.

We repeated the same permutation procedure to determine the individual significance for the nERP related to muscle sensor, the PD signal, as well as the breakdown of the AC signal from each channels and wavelength separately. Finally, the recorded ECG signal was subjected to the same procedure as with the NIRS data with the exception that no correction for the different sampling rates was necessary as the EEG and ECG signal were recorded by the same device. Group statistics compared the correlation coefficients from the different measures to one another, e.g. AC signal between brain and muscle, using a series of standard paired T-tests.

## Additional Information

**How to cite this article**: Mensen, A. *et al*. The occurrence of individual slow waves in sleep is predicted by heart rate. *Sci. Rep*. **6**, 29671; doi: 10.1038/srep29671 (2016).
